# Implementation and Outcomes of a Train-the-Trainer Program at Behavioral Health Treatment Centers as a Mechanism to Maintain Organizational Capacity to Address Tobacco Use Disorder

**DOI:** 10.3390/ijerph182111635

**Published:** 2021-11-05

**Authors:** Vijay Nitturi, Tzuan A. Chen, Isabel Martinez Leal, Virmarie Correa-Fernández, Kelli Drenner, Bryce Kyburz, Teresa Williams, Ezemenari M. Obasi, Maggie Britton, Molly Howard, Rogelio Rangel, Jeni Sharp, Shelley Smith, Lorraine R. Reitzel

**Affiliations:** 1 Department of Psychological, Health, and Learning Sciences, University of Houston, Houston, TX 77204, USA; vnitturi@central.uh.edu (V.N.); tchen3@central.uh.edu (T.A.C.); imarti31@central.uh.edu (I.M.L.); vcorreaf@central.uh.edu (V.C.-F.); kldrenne@central.uh.edu (K.D.); emobasi@central.uh.edu (E.M.O.); mkbritto@central.uh.edu (M.B.); 2 HEALTH Research Institute, University of Houston, Houston, TX 77204, USA; 3 Integral Care, Austin, TX 78703, USA; bryce.kyburz@integralcare.org (B.K.); teresa.williams@integralcare.org (T.W.); 4 Heart of Texas Region MHMR, Waco, TX 76701, USA; molly.howard@hotrmhmr.org (M.H.); Jeni.Sharp@hotrmhmr.org (J.S.); 5 Border Region Behavioral Health Center, Laredo, TX 78041, USA; rogelior@borderregion.org; 6 West Texas Centers, Big Springs, TX 79720, USA; shelley.smith@wtcmhmr.org

**Keywords:** tobacco cessation, train-the-trainer, behavioral disorders, mental health disorders, education

## Abstract

Despite prior successful implementation of Taking Texas Tobacco Free (TTTF), an evidence-based tobacco-free workplace program, in local mental health authorities (LMHAs), post-implementation employee attrition necessitated continuing education on tobacco-free policies and tobacco treatment practices. Here, we report on the outcomes of a train-the-trainer program which trained “champions” to deliver tobacco cessation education at their LMHAs. Three LMHAs participated in program implementation via 10 champions, iteratively trained and coached by TTTF. Measures administered evaluated four goals: (1) increase champions’ self-efficacy in delivering trainings, (2) achieve program fidelity via TTTF staff evaluation of trainer effectiveness and knowledge increases among attending employees, (3) achieve stakeholder program acceptability, and (4) achieve program adoption via an increase in follow-up trainings. Champions’ self-efficacy increased throughout TTTF training. TTTF staff ratings of champion-led trainings met the targeted range for trainer effectiveness; employees had a 28.71% knowledge increase over baseline post-training (*p <* 0.001). Employees rated champions’ training delivery “very good” to “excellent”, on average; both champions and employees were, on average, “satisfied” to “extremely satisfied” with the curriculum and training received. There was an increase over baseline in trainings delivered during follow-up, and trainings increased in length and topic coverage. Ultimately, the train-the-trainer program achieved the intended goals, although not all changes were statistically significant, likely at least partially attributable to small sample sizes. Overall, these results suggest that TTTF’s train-the-trainer program was successful in its delivery and intention to build capacity for the provision of in-house tobacco education trainings to behavioral health employees/providers. However, further evaluation in additional settings, with more champions, et cetera, is necessary to validate these findings, ensure their replicability, link program implementation with reduced patient tobacco use rates, and assess long-term sustainability.

## 1. Introduction

Tobacco use remains the number one cause of preventable death and disability within the United States [1]. Although tobacco use has decreased due to decades of public health interventions, it remains a significant issue within groups that suffer from behavioral health conditions (e.g., individuals with mental health, and/or substance use disorders), who smoke cigarettes at higher rates than the general population [1,2,3]. For example, while ~14% of adults in the United States are current smokers, the rate of smoking amongst adults with behavioral health conditions is 23% [4]. In adults with three or more behavioral health conditions, the smoking rate almost triples (~61%) [2]. These statistics ultimately explain why half of the total deaths resulting from tobacco use, almost 200,000 people each year, come from adult populations with behavioral health conditions [2]. As a result, individuals with behavioral health conditions are identified as a tobacco health disparities group, and tobacco use within this community urgently needs to be addressed [3,5,6].

Despite this alarming evidence, according to the most comprehensive national evaluation conducted to date, only 48.9% of behavioral health treatment centers screened for tobacco use in 2016, and only 25.2% of centers offered nicotine replacement therapy [7]. Furthermore, smoke-free workplace policies were in effect in less than half of these settings (48.6%) [7]. Public-private partnerships such as the Substance Abuse and Mental Health Services Administration and the Smoking Cessation Leadership Center have made progress on this issue, providing workshops, talks, and technical assistance to and establishing collaborative initiatives between organizations promoting tobacco cessation [8]; however, behavioral health facilities are still resistant to the idea of incorporating evidence-based policies and procedures to address tobacco use disorder amongst their patient stakeholders [9]. There are several reasons for this hesitation. Many providers working with individuals with behavioral health conditions are resigned to patients’ high smoking rates [3] and assume that tobacco cessation will negatively impact treatment of the behavioral health conditions [10]. However, research has shown that just the opposite is true: tobacco cessation, in the long term, improves mental health and non-nicotine substance dependence recovery outcomes [7,11]. Providers may also lack the necessary training to administer evidence-based nicotine dependence care and tend to place a low emphasis on addressing tobacco use disorder compared to other clinical goals [9,12,13]. Programs to address high rates of cigarette smoking need to be implemented in behavioral health treatment settings whereby screening for and treating tobacco use becomes a part of standard/routine clinical practice.

Taking Texas Tobacco Free (TTTF) is an evidence-based, multi-component tobacco control program designed to build healthcare agencies’ organizational capacity to address tobacco use with evidence-based policies and practices. The current study focuses on the education components of the TTTF program that include provision of tobacco education and training for both non-clinical staff and clinical providers [14]. To address the problem of high rates of tobacco use among adults with behavioral health conditions, TTTF partnered with 23 local mental health authorities (LMHAs) for the implementation of the program (see [12,13,14,15,16,17] for more information about this overarching program, which ended in 2018). LMHAs are non-profit, state-supported, geographically-organized, behavioral health organizations providing services to Texans within a varying number of clinics embedded within each service area. Evaluations of the TTTF program cited knowledge gains in both providers and non-clinical employees as a result of training efforts [12], and increases in empirically-based clinician behaviors to address tobacco use with their patients [15,18,19,20].

TTTF worked closely with “champions”, who were employees appointed by center leadership to oversee and ensure the success of program implementation as part of their workplace responsibilities. Champions were supported to attend Certified Tobacco Treatment Specialist training and were tasked with being a long-term (post-funding) internal resource to their colleagues about treating tobacco use disorder, to include leading subsequent internal employee trainings on the topic (e.g., annual trainings, in-service trainings, new employee trainings) [14]. Unfortunately, participating behavioral health treatment center stakeholders reported two main challenges to the sustainability of the educational components of the TTTF program: (1) employee turnover whereby TTTF champions left their agency, and (2) low rates of subsequent champion-initiated employee trainings (potentially and anecdotally attributable to low self-efficacy to do so). In response, the TTTF team applied for and received an award from the Cancer Prevention and Research Institute of Texas to build the capacity of LMHA partners’ in-house tobacco trainings through a provided curriculum and train-the-trainer program. This program was intended to address the sustainability of in-house employee trainings on the harms of tobacco use and how to address them with evidence-based interventions by: (1) creating a flexible curriculum, adaptable to individual center’s needs, for agency-led trainings; (2) engaging >1 champions at participating LMHAs to train them on the curriculum and coach them on their delivery of it; and (3) providing technical assistance on how to best establish a long-term training initiative within their agencies. These strategies were intended to not only increase champions knowledge on tobacco use and treatment, but also to solidify their self-efficacy to disseminate that knowledge to others via formal in-house trainings.

The current study describes the implementation and outcomes associated with TTTF’s “train-the-trainer program”, which can serve as a model for wider dissemination and implementation in behavioral health treatment settings.

## 2. Materials and Methods

### 2.1. Organizational Participant Recruitment

Recruitment for the current study was accomplished via an email invitation addressed to each LMHA Chief Executive Officer (CEO) who provided a letter of support to accompany the grant application to conduct this work. Of the 5 LMHAs providing letters of support (in 2019), each of which had participated previously in the overall TTTF comprehensive tobacco-free workplace implementation [14], 3 ultimately accepted the post-funding invitation (in 2020) to actively participate in the train-the-trainer program’s implementation. The 2 LMHAs that declined to participate cited conflicting demands that would prohibit their participation. See Supplemental Table S1 for demographics of participating LMHAs with information provided by the LMHA CEO from the prior years’ annual report. The 3 LMHAs who chose to participate in the current program completed the overall TTTF comprehensive tobacco-free workplace implementation on 10/4/2017 (LMHA 1), 4/5/2016 (LMHA 2), and 8/12/2015 (LMHA 3).

Written consent for participation was obtained from participating LMHA leadership prior to study participation via a memorandum of understanding. Program implementation spanned the fall of 2020 through the spring of 2021 with start and end dates varying by participating LMHA based on their preferences and progress. All procedures were approved by the Institutional Review Board of the University of Houston (STUDY00002164 beginning 8 April 2020).

### 2.2. Participating Providers (Also Known as Champions)

The LMHA CEO and/or their designee selected the treatment providers who would participate in this project as part of their workplace responsibilities. The team encouraged, to the extent possible, that the champions for the larger/prior comprehensive workplace program administration [13,14,16,17] and/or those who had previously attended Certified Tobacco Treatment Specialist Training be selected as the participating providers (also known as champions) of the current effort. However, this was only possible in 2 of the 10 cases due to losing several additional Certified Tobacco Treatment Specialist trained clinicians across these LMHAs to turnover. Champions provided consent for participation and data collection. They provided information about their experience with training, their self-ratings of their training capacity prior to train-the-trainer implementation, and the number and duration of tobacco trainings conducted in their LMHA during the year prior to program implementation (not specific to training conducted by themselves, but rather training conducted within the LMHA overall). Champions noted that the only tobacco trainings provided were to new employees during their onboarding process and occurred approximately monthly within the organization. See Table 1.

### 2.3. Program Implementation

**Figure 1:**
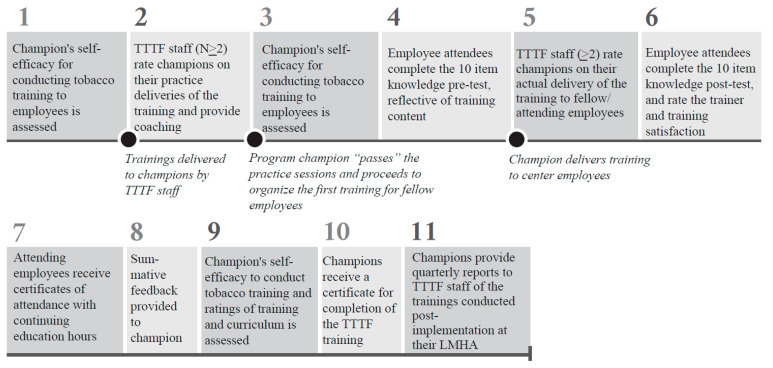
This infographic summarizes the implementation steps of the train-the-trainer program. Important events have been marked in between certain steps. TTTF = Taking Texas Tobacco Free.

The train-the-trainer program was implemented in a series of steps (see Figure 1 for a summary). The implementation period ranged from 4 months to 6 months between participating LMHAs. Given COVID 19-related concerns, all training activities, originally designed to be delivered in-person, were conducted live, via Microsoft Teams and/or Zoom, to allow interaction and discussion between trainers and trainees, and all data collection was accomplished electronically via Qualtrics or email. Following a self-assessment of champions training self-efficacy (Step 1; see Figure 1), TTTF provided a 4–5 h comprehensive training to the champions on tobacco use and treatment for individuals with behavioral health treatment needs. On a separate occasion, TTTF staff next delivered a scaled down, 90-min version of the training to reinforce knowledge and provide champions a model of the training they would conduct. Thereafter, the TTTF staff observed the champions practice deliver the 90-min presentation twice (on separate occasions) to no audience or to a small audience of other champions. Each time, champions were provided oral and written (i.e., rater forms) feedback from TTTF staff. Champions could not proceed to training LMHA employees (i.e., actual trainings) until they had reached “very good” or “excellent” ratings by TTTF observers on delivery of the training curriculum and effectiveness as a trainer. Various tips were presented throughout the training process, along with coaching on how to improve presentation (Step 2). Prior to actual training delivery, champions again rated their training self-efficacy (Step 3). Champions then delivered 2 actual trainings to a group of their LMHA’s current employees, all of whom were either highly encouraged or mandated to attend. Attending employees completed a pre-training knowledge test (Step 4) and attending TTTF staff provided ratings of the champion’s delivery (Step 5). After the training, employees completed a post-training knowledge test and provided ratings of the champion’s performance and their satisfaction with the training itself (Step 6). Attending employees received a certificate (Step 7) and summative feedback was provided to champions in written form that included a summary of knowledge gained by attending employees, as well as TTTF staff and employee ratings of the training (Step 8). Thereafter, the champions again rated their training self-efficacy, and their satisfaction with their training experience with TTTF and the provided curriculum (Step 9). Champions then received a program completion certificate from TTTF (Step 10). This marked the end of the program implementation, after which data were collected quarterly from the champions with regard to additional employee trainings provided (Step 11).

### 2.4. Program Goals

The goals and objectives of the program were as follows: (Goal 1) increase champions’ self-efficacy for delivering tobacco education throughout our training program; (Goal 2) evaluate program fidelity by achieving (a) team observer ratings of “very good” to “excellent” in the delivery of the education to the employees by the trained champions, and (b) achieve ≥20% knowledge increase among employee attendees from the delivered trainings; (Goal 3) evaluate program acceptability by achieving (a) employee attendees’ ratings of “very good” or “excellent” for the effectiveness of the trainer and ratings of “satisfied” or “extremely satisfied” with the training provided by the champion, and (b) champion ratings of “satisfied” or “extremely satisfied” with the curriculum and their training to implement it; and (Goal 4) evaluate program adoption via an increase over baseline in the number of education sessions delivered to employees by the champions post-program implementation.

### 2.5. Measures of Relevance

#### 2.5.1. Champion Self-Efficacy Self-Assessments (Goal 1, Increase Self-Efficacy)

Champions completed a 10-item face-valid, investigator-generated survey administered online to assess changes in their training self-efficacy over time; this was completed (1) prior to undergoing TTTF-led training; (2) post-training but prior to the champion-led delivery of the training to employee attendees; and (3) post the champion-led employee trainings. See Figure 1. Items on the survey captured elements regarding mastery, vicarious experience, emotional state, and verbal persuasion, all constructs theorized to influence an individual’s self-efficacy [21]. The following are example items: “I currently have the capacity to deliver trainings in tobacco control,” “When conducting a training, I am afraid attendees will notice that I am nervous,” and “I have received support and encouragement to engage in activities as a trainer/health educator.” Each of these items was rated on a 5-point scale where 1 = strongly disagree and 5 = strongly agree. The final item assessed overall self-efficacy (“Overall, how would you rate your capacity to conduct a training on tobacco control to members of your organization?”) on a 5-point scale where 1 = poor and 5 = excellent. This item was meant as a summary of overall self-efficacy and was used in the assessment of Goal 1 (increase self-efficacy over time).

#### 2.5.2. TTTF Staff Assessments of Champions’ Training Delivery (Goal 2a, Fidelity)

TTTF staff used a 17-item face-valid, investigator-generated survey to rate the champions’ delivery of the training during “practice” presentations and two “actual” employee trainings. An example item was, “The trainer appeared confident and comfortable with the material,” with ratings from 1 = strongly disagree through 5 = strongly agree. These items were meant to provide feedback to the champion about areas for improvement. The 2 final items were intended to summarize performance and read: “Overall, how would you rate the delivery of the training curriculum by the trainer to setting stakeholders?” and “Overall, how would you rate the effectiveness of the trainer as a teacher?” on a 5-point response scale where 1 = poor through 5 = excellent. Champions had to achieve ratings of “very good” (4) or “excellent” (5) by each observing TTTF staff member in practice presentations to progress on to train employees; thus, Goal 2a for fidelity was measured with each of the final 2 items as rated by TTTF staff for the presentations the champions gave to actual employee trainees. The goal was that TTTF staff would rate the delivery of these actual trainings, on average, as falling between “very good” and “excellent”.

#### 2.5.3. Employee Attendee Assessments of Knowledge Gain (Goal 2b, Fidelity)

Knowledge gain from the champion-delivered training was collected before and after training using a 10-item face valid, investigator-generated knowledge test with items directly reflecting training content. In each case, a champion emailed the knowledge test link to employees who signed up for the training to complete within the week prior to the training, and then emailed a link to the post-training knowledge test immediately following training, with a request to complete it as soon as possible, preferably within the same day but at most within 7 days. Employees were instructed to complete the tests without reference to training slides, the internet, etc. Items on both the pre- and post-training knowledge tests were the same; however, no feedback on pre-test accuracy was presented to the test taker, in an attempt not to bias responses to the post-training knowledge test. An example item was, “Which of the following is NOT one of the “5As” or tobacco cessation brief intervention?” with answer options (a) ask; (b) arrange; (c) assess; and (d) allow. This measure has been used in our prior work [12,15,22,23,24]. No personally identifying information about the employee was collected on this survey at either administration. Goal 2b for fidelity was measured by the percent increase in employee knowledge from pre-to post-training. Based on prior work, we aimed to achieve a statistically significant knowledge gain of ≥20% on average between all employee attendees.

#### 2.5.4. Employee Attendee Assessments of Champion-delivered Trainings (Goal 3a, Program Acceptability)

Employee attendees rated the champion on the delivered trainings using a 16-item face-valid, investigator-generated survey administered online. The first 14 items assessed different aspects of the trainer and the presentation, with items such as: “The training was engaging”, “The trainer’s articulation and voice level were clear”, and “The trainer seemed well-prepared for the training”, that were rated on a 5-point scale where 1 = strongly disagree and 5 = strongly agree. These items were meant to provide feedback to the champion about areas of strength as well as areas for improvement. The other 2 items were: (1) “Overall, how would you rate the effectiveness of the trainer as a teacher?” (1 = poor through 5 = excellent); and (2) “Overall, please rate how satisfied you were with this training”, (1 = extremely dissatisfied through 5 = extremely satisfied). These 2 final items measured program acceptability from the employee attendees’ perspective (Goal 3a). The goal was for employee attendees, on average, to rate the trainer effectiveness as falling between “very good” and “excellent” and that, on average, they were between “satisfied” and “extremely satisfied” with the training content. No personally identifying information about the employee attendee was collected on this survey.

#### 2.5.5. Champion Ratings of TTTF-Provided Curriculum and Training (Goal 3b, Program Acceptability)

Champions were asked 2 questions about the materials and instruction the train-the-trainer program provided them: (1) “Overall, please rate your satisfaction with curriculum provided to you by the TTTF team”, and (2) “Overall, please rate your satisfaction with the training you received to implement the curriculum provided to you by the TTTF team”, with response options ranging from 1 = extremely dissatisfied and 5 = extremely satisfied. These items measured program acceptability from the champions’ perspective (Goal 3b).

#### 2.5.6. Champions’ Post-Implementation Quarterly Reports (Goal 4, Adoption)

Champions provided TTTF with the number and duration of trainings delivered following successful completion of the train-the-trainer program, which is how Goal 4 (adoption) was assessed. This information was gathered quarterly for each LMHA post-program completion on a form via email. LMHA 1 provided 2 quarters (6 months), LMHA 2 provided 3 quarters (9 months), and LMHA 3 provided 2 quarters (6 months) of post-implementation training information. The different follow-up periods were based on when each LMHA finished the program and the consequent time remaining in the grant follow-up data collection period.

### 2.6. Statistical Analysis

Goal 1 (increase champions’ self-efficacy over time) was assessed using Wilcoxon signed-rank tests to test the changes over time within each champion. This test was used because there were few observations. Goal 2, Objective 2a (evaluate program fidelity by TTTF staff assessments of champions’ training delivery to employees) was assessed with descriptive statistics, and Objective 2b (assess fidelity by employee knowledge gain) was assessed by calculating the percent increase in knowledge within training session, with an analysis of covariance using employee as the analysis unit (controlling for champion) assessing statistical significance of the change. The formula for the percent increase in knowledge is as follows:

(1) % increase=[(post/pre)−1] ∗ 100%

Goal 3, Objective 3a (acceptability of champion-led training as reported by attending employees), and 3b (acceptability of TTTF training and curriculum) were assessed with descriptive statistics. Finally, Goal 4 (adoption) was calculated numerically by LMHA. Because the number of trainings occurring pre-implementation was assessed over a 12-month period, and the number of trainings conducted post-implementation was assessed over varying numbers of quarters, the assessment of this goal was achieved by dividing the number of trainings by the number of months assessed. The percent increase in trainings offered from before to after program implementation was calculated with equation 1.

Alpha, for goals that used statistical comparisons of change over time (Goal 1, Goal 2b), was set at 0.05. All analyses were conducted using SAS 9.4 [25].

## 3. Results

### 3.1. Champions’ Changes in Self-Efficacy (Goal 1)

Table 2 presents the descriptive statistics including means and standard deviations for each of the LMHAs and all participating LMHAs combined. The means of total self-efficacy across all champions over time were 2.80 (SD = 1.32; Median = 3, IQR = 1 to 4), 3.60 (SD = 0.52; Median = 4, IQR = 3 to 4), and 3.90 (SD = 0.57; Median = 4, IQR = 4 to 4) on a five-point scale. Our goal of increasing champions’ self-efficacy for delivering tobacco education over time throughout our training was achieved, but the results from Wilcoxon signed-rank tests revealed that changes were not statistically significant across time overall (*S* = 7.5, *p* = 0.0625) or by LMHA (*S*: 0.5–1, *p* ≥ 0.5000).

### 3.2. TTTF Staff Assessments of Champions’ Training Delivery (Goal 2a, Fidelity)

Overall, champions received between 1 and 3 “practice” trainings with TTTF staff observers (*n* = 8 champions completed 1 practice training; *n* = 2 champions completed 3 practice trainings). All champions successfully received ratings of 4 “very good” or 5 “excellent” on their delivery of the training curriculum and on their effectiveness as a trainer.

Table 3 shows the mean/median TTTF staff ratings of champions by LMHA in the delivery of the education to the employees. All LMHA ratings fell between “very good” and “excellent,” meeting this program goal.

### 3.3. Employee Attendee Assessments of Knowledge Gain (Goal 2b, Fidelity)

Table 4 shows that the employee attendees exhibited a 28.71% increase in knowledge overall. Results of analysis of covariance indicated that knowledge gain was significant overall (*F* = 130.97, *p* < 0.001) and by LMHA (*F*: 9.29–83.75, *p’s* ≤ 0.003). While overall knowledge gain exceeded the program goal of ≥20% and was statistically significant at each LMHA, it is worthy of note that LMHA 2′s knowledge gain was <20%.

### 3.4. Employee Attendee Assessments of Champion-delivered Trainings (Goal 3a, Acceptability)

Table 5 displays the acceptability of champion-led training as reported by attending employees. The mean ratings were between “very good” and “excellent” (trainer effectiveness) and between “satisfied” and “extremely satisfied” (training content), indicating achievement of this program goal.

### 3.5. Champion Ratings of TTTF-Provided Curriculum and Training (Goal 3b, Acceptability)

Table 6 shows the acceptability of the program curriculum and training provided to champions. The curriculum and training provided by TTTF were highly rated by the champions, indicating the achievement of this program goal.

### 3.6. Program Adoption (Goal 4)

TTTF records indicated that LMHA 1 conducted 6 trainings for 30 employees across 2 follow-up quarters, LMHA 2 conducted 16 trainings for 135 employees across 3 follow-up quarters, and LMHA 3 conducted 7 trainings for 57 employees across 2 follow-up quarters. Relative to baseline, this represents a 35.02% increase in the number of education sessions delivered to employees by the champions post-training. Education sessions typically lasted 90 min and were administered to new and old employees alike, a stark contrast to the pre-champion training sessions which mostly lasted 10 min and were only given to new employees.

## 4. Discussion

This study described the implementation and outcomes of a train-the-trainer program aimed at building capacity for in-house tobacco education provision to employees by designated champions at behavioral health treatment centers 4–6 years following their participation in a comprehensive tobacco-free workplace program. Each program goal for this train-the-trainer program was met. Overall, these results suggest that TTTF’s train-the-trainer program was successful in its delivery and intention to build capacity for the provision of in-house tobacco education trainings to behavioral health employees/providers. Future work will need to assess the impacts of this program on the sustainment of all evidence-based tobacco use policies and practices as associated with a comprehensive tobacco-free workplace program (e.g., compliance with the tobacco-free workplace policy, provision of tobacco screening and treatment to patients), which was beyond the scope and timeline of the underlying grant. However, results speak to the potential upkeep of one major component of the TTTF comprehensive tobacco-free workplace program: the provision of employee education about addressing tobacco use amongst their behavioral health patients, which has previously been associated with increased screening and treatment of tobacco use [15,22,23,24]. An important future direction of this work is to evaluate whether TTTF’s train-the-trainer program’s implementation was linked with clinician behavior change and, ultimately, reduced patient tobacco use rates.

Programs such as this, which create opportunities for cascade training within an organization and that empower employee stakeholders to be the “experts” in tobacco use cessation continuing education, are vital for implementation in behavioral health settings given that a lack of education on these issues has been cited by providers as a major impediment to providing tobacco cessation care [9,12,13]. This is especially the case in settings that experience high rates of employee turnover [26], such as LMHAs. The TTTF staff provided a step-by-step program train-the-trainer implementation guide [27] and all evaluation materials to the participating LMHAs to enable them to replicate the training with new champions over time. However, longer follow-up periods are needed to ensure that LMHAs train new champions when old ones leave the organization, so that turnover does not reduce the LMHAs’ tobacco training capacity. Although the train-the-trainer program, available online [27], may be helpful for sustainment of educational efforts in behavioral health settings following a comprehensive tobacco-free workplace program implementation, future research should address ways that it can be incorporated as part of comprehensive programming implementation or as a stand-alone component to increase tobacco use disorder intervention with patients. Moreover, longer periods of follow-up are necessary to ensure that program adoption gains are sustained over time, even in the absence of staff turnover.

The processes and evaluation of TTTF’s train-the-trainer program were aligned with other train-the-trainer programs and included the measurement of champion self-efficacy for training (Goal 1), the assessment of program fidelity through employee knowledge gain (Goal 2b), and the acceptability of the program’s curriculum by the champions (Goal 3b) [28,29,30,31,32,33,34,35]. However, this study also expands the train-the-trainer literature. Unlike several recent health-focused train-the-trainer program studies (including two programs specifically addressing tobacco cessation training), the current work was the only one to assess the fidelity of the training program by having those who conducted the program observe their respective champion-led trainings [28,29,30,31,32,33,34,35]. Most train-the-trainer programs relied on evaluating fidelity by measuring employee knowledge gain through assessments, which may present a limited picture of trainer effectiveness [28,29,30,32,34]. Moreover, of the two known tobacco cessation train-the-trainer program publications, only one assessed employee knowledge, but only post-training [30]. Additionally, staff observation of training sessions with employees (the other measure of fidelity in the current work) and the written summative report provided to the champions provided fodder for subsequent improvements in delivery.

Another potentially unique aspect of the current work relative to the literature was the sustainment follow-up, albeit for a limited time, which was only evident in three of the eight train-the-trainer programs identified in the literature [28,31,35], one of which simply asked their trainees whether they would be likely to keep training others in their field [35]. Given that continued dissemination of knowledge through center-led training is the ultimate goal of train-the-trainer programs, the collection of data regarding trainings conducted once the treatment center is “on its own” and no longer partnering with an outside training program is important. Furthermore, our evaluation of acceptability was also relatively unique, as only four out of eight train-the-trainer programs—only one of which was focused on tobacco training–evaluated the acceptability of champion-led trainings by their trainees [29,30,31]. This is also important to measure, as trainee engagement and favorable perception of the training may facilitate greater knowledge gains and may help in the promotion of future training programs for others in their field.

It is important to acknowledge that not all results of TTTF’s train-the-trainer program were statistically significant. Namely, although the average champion self-efficacy increased from a largely “good” baseline (2.80) to an outcome approaching “very good” (3.90), these improvements were not statistically significant. This inability to detect a significant finding was likely a function of the study being underpowered (N = 10). A sample size of 35 would be needed to achieve an adequate power of 80%, with practical significance of *d* = 1.1 implying a large effect size (see [36] for reference). However, our program goal was not to achieve statistical significance here, but rather to achieve increases over time that may have practical significance for training capability sustenance. Moreover, high baseline self-efficacy scores detailed in Table 1 and Table 2 may have resulted in a ceiling effect given the five-point Likert scale that was used. These high baseline scores are potentially a product of most champions having had either previous specialized training in tobacco intervention and/or a significant history of providing trainings (see Table 1), which is supported by no increases in self-efficacy being reported in another tobacco train-the-trainer program where there were strict tobacco-related knowledge and previous training criteria to be selected as a trainer [30]. Furthermore, in a different tobacco train-the-trainer program that included trainers with lower levels of tobacco knowledge and previous training, there were self-reported significant increases (over baseline) in ability to teach tobacco cessation [31]. However, it may be desirable to train knowledgeable and motivated individuals (as opposed to a naiver group) to prevent high levels of attrition. Despite that the current evaluation of this program was favorable, additional work is needed with larger samples, additional settings, et cetera, to evaluate the generalizability of the current results.

It may also be worth noting that although the provision of post-implementation training during the follow-up period showed only a modest increase relative to pre-implementation, the nature of the trainings differed in both intended targets (any employee versus new employees only) and training duration (90 min versus 10–60 min), which likely also increased the depth or breadth of relevant topics covered. The TTTF curriculum included information on the prevalence of smoking amongst behavioral health patients, the benefits of quitting on their mental health and quality of life, how to screen for and intervene on tobacco use with recommended pharmacotherapies and dosages, and the reasons for the tobacco-free workplace policy and how to address violators. Thus, it was fairly comprehensive in its coverage of multi-level, empirically-supported tobacco control strategies that had been part of the comprehensive tobacco-free workplace program previously implemented years before. It is possible that more comprehensive trainings are associated with greater long-term compliance with tobacco-free policies and cessation treatment provision; however, this needs to be assessed in future work.

Results should be considered in terms of limitations and strengths. One limitation is a low number of participating LMHAs (*n* = 3) and champions (*n* = 10); however, program participants were commensurate with the underlying funding mechanism and funder-approved recruitment goals. Furthermore, certain assessments (e.g., knowledge, ratings of trainers) were completed by employees, for which there were larger sample sizes (N = 197–265). Despite this, future research should replicate this program using a greater number of participating behavioral health centers and champions to facilitate the evaluation of statistically significant changes. A second limitation is that we do not know the extent to which this program is adaptable outside of the specific behavioral health settings in which it took place. Future research should validate the impact of this program outside of the behavioral health settings as well as outside of Texas, especially given its possible utility across different treatment settings (e.g., substance use treatment programs, Federally Qualified Health Centers) and states. For example, different degrees and areas of staff training might be necessary in states with existing policies around tobacco use within addiction treatment settings, such as New York [37,38]. A third limitation is that we did not assess any clinically relevant outcomes, such as the impact this program had on patient tobacco use rates. This is an important aspect to assess in the future given research suggesting increases in tobacco use screening and treatment provision following training [15,22,23,24] may ultimately decrease tobacco use rates, thus ameliorating health disparities amongst individuals with behavioral health conditions.

One strength of this project is that an additional project goal included developing a step-by-step implementation guide available online for broad passive dissemination [27], which was accomplished during the funding period. Thus, we have detailed the specifics of this program and made it freely and widely available to researchers and behavioral health centers alike. Future work should focus on the extent to which results of this implementation generalize to behavioral health settings that implement this program according to the implementation guide, but with minimal to no technical assistance from the TTTF staff. An additional strength of this implementation was its delivery via virtual means due to COVID-19. Although the grant was written with in-person procedures, the execution of the project once funded needed to shift for the safety and comfort of the TTTF team and its LMHA stakeholders. However, this fortuitous switch in procedures yielded advantages in convenience (especially for our rural stakeholders, with whom we could more frequently engage virtually), cost, as well as opportunities to practice using technology with participating LMHAs. Ultimately, this may have also made the step-by-step implementation guide more practical for systems-based implementation as well.

## 5. Conclusions

One reason cited for why tobacco use is not regularly addressed in behavioral health/addiction treatment settings is a lack of clinician knowledge/training on the topic [39,40,41,42]. Moreover, annual trainings may be insufficient to lead to sustained systemic change in these treatment settings due to high turnover rates [26]. Consequently, there is a need for novel training programs that can build and sustain in-agency capacity for addressing the high tobacco use rates amongst their stakeholders. The TTTF train-the-trainer program was designed to build capacity for in-house tobacco education provision to employees by designated employee champions at behavioral health treatment centers, four to six years following their LMHA’s participation in a comprehensive tobacco-free workplace program. Implementation of the train-the-trainer program resulted in increased self-efficacy for the delivery of the curriculum as reported by the participating champions, was implemented with fidelity to the provided curriculum, was acceptable to the champions delivering the trainings and attending LMHA employees alike, and increased the number of educational sessions delivered post-program implementation while extending training length, depth, and breadth and targeted employees. These findings provide an important foundation for future train-the-trainer programs targeting tobacco use in behavioral health settings. However, further evaluation in additional settings, with more champions, et cetera, is necessary to validate these findings, ensure their replicability, link program implementation with reduced patient tobacco use rates, and assess long-term sustainability.

## Figures and Tables

**Table 1:** Participating Local Mental Health Authorities and Champions’ Pre-Program Implementation Training Information.

	New Employee Tobacco Trainings Conducted Annually (Duration in Minutes)	Tobacco Training Experience (In Months/SD)	Other Training Experience (In Months/SD)	TTTF PC by History and/or CTTS-trained?	PC Self-Rating of Self-Efficacy to Conduct Tobacco Training
LMHA 1	12 (10 m)	39.75 (55.93)	192 (114.81)		
PC1		3	279	No	very good
PC2		36	69	No	very good
PC3		0	300	No	good
PC4		120	120	No	good
LMHA 2	12 (60 m)	2 (3.46)	92.33 (90.92)		
PC1		0	60	No	good
PC2		0	195	No	poor
PC3		6	22	Yes	very good
LMHA 3	11 (10 m)	24 (41.57)	80 (84.07)		
PC1		72	174	Yes	very good
PC2		0	54	No	poor
PC3		0	12	No	poor

Note. PC = program champion; CTTS = Certified Tobacco Treatment Specialist Training; SD = standard deviation. Champions’ self-ratings of capacity to conduct tobacco training were on the following scale: 1 = poor, 2 = fair, 3 = good, 4 = very good, 5 = excellent.

**Table 2:** Champions’ Self-reported Self-efficacy by Local Mental Health Authorities Over Time.

LMHA	N	Before TTTF Training	Pre-Employee Training	Post-Employee Training
		Mean (SD)/Median [IQR]		
All LMHAs	10	2.80 (1.32)/3 [1–4]	3.60 (0.52)/4 [3, 4]	3.90 (0.57)/4 [4]
LMHA 1	4	3.50 (0.58)/3.5 [3, 4]	3.50 (0.58)/3.5 [3, 4]	4.00 (0.82)/4 [3.5–4.5]
LMHA 2	3	2.67 (1.53)/3 [1–4]	3.67 (0.58)/4 [3, 4]	3.67 (0.58)/4 [3, 4]
LMHA 3	3	2.00 (1.73)/1 [1–4]	3.67 (0.58)/4 [3, 4]	4.00 (0.00)/4 [4]

Note. None of the differences were significant at the level of 0.05. Self-efficacy was measured with a single item. “Overall, how would you rate your capacity to conduct a training on tobacco control to members of your organization?” on a 5-point scale where 1 = poor, 2 = fair, 3 = good, 4 = very good, 5 = excellent. LMHA = local mental health authority. N = number of participating champions. SD = standard deviation. IQR = interquartile range.

**Table 3:** TTTF Staff Assessments of Champions’ Tobacco Education Training Delivery to LMHA Employees.

LMHA			“Overall, How Would You Rate the Delivery of the Training Curriculum by the Trainer to Setting Stakeholders?”	“Overall, How Would You Rate the Effectiveness of the Trainer as a Teacher?”
	Unique TTTF Staff Observers (N)	Champions(N)	Mean (SD)/Median [IQR]	
All LMHAs	6	10	4.53 (0.43)/4.55 [4, 5]	4.56 (0.54)/4.78 [4, 5]
LMHA 1	4	4	4.63 (0.48)/4.75 [4.25–5]	4.56 (0.72)/4.88 [4.13–5]
LMHA 2	4	3	4.15 (0.31)/4 [3.94–4.5]	4.17 (0.29)/4 [4–4.5]
LMHA 3	4	3	4.78 (0.20)/4.75 [4.6–5]	4.93 (0.12)/5 [4.8–5]

Note. The scale for these items was 1 = poor, 2 = fair, 3 = good, 4 = very good, 5 = excellent. LMHA = local mental health authority. N = number. SD = standard deviation. IQR = interquartile range. Unique TTTF staff observers= the number of unique TTTF staff observers of the training delivery. Three TTTF staff observed champions’ training delivery in all 3 LMHAs, and each of the other 3 TTTF staff observed champions’ training delivery in only 1 of the 3 LMHAs. Two TTTF staff observed each training, except for one training, where 3 TTTF staff observed.

**Table 4:** Knowledge Increase among Attending Employees of the Champion-delivered Trainings.

	Pre-Training Score		Post-Training Score		Pre vs. Post
LMHA	N	Mean (SD)	N	Mean (SD)	Increase %
All LMHAs ***	265	6.27 (1.83)	201	8.07 (1.48)	28.71%
LMHA 1 ***	138	6.10 (1.68)	112	8.01 (1.61)	31.31%
LMHA 2 **	65	6.57 (2.11)	36	7.78 (1.46)	18.42%
LMHA 3 ***	62	6.32 (1.80)	53	8.40 (1.17)	32.91%

Note. ** *p* < 0.01; *** *p* < 0.001. Knowledge gain was assessed with a face-valid, investigator-generated knowledge test with 10 items; the possible range of knowledge was 0 to 10 at each administration. LMHA = local mental health authority. N = number of employee attendees providing pre- or post-training tests. SD = standard deviation; the number of attendees post-training knowledge test answers was less than those providing pre-training knowledge test answers due to some employees being unable to attend the training despite signing up, employees needing to leave training early due to work demands, and employee preference not to participate in this aspect of data collection.

**Table 5:** Local Mental Health Authority Employee Attendee Assessments of Champion-delivered Trainings.

		“Overall, How Would You Rate the Effectiveness of the Trainer as a Teacher?” ^a^		“Overall, Please Rate How Satisfied You Were with this Training” ^b^	
LMHA		N	Mean (SD)	N	Mean (SD)
All LMHAs		199	4.28 (0.83)	197	4.33 (0.64)
LMHA 1		110	4.08 (0.91)	108	4.17 (0.65)
LMHA 2		36	4.44 (0.69)	36	4.61 (0.49)
LMHA 3		53	4.57 (0.60)	53	4.47 (0.61)

Note. ^a^ The scale for this item was 1 = poor, 2 = fair, 3 = good, 4 = very good, 5 = excellent; ^b^ the scale for this item was 1 = extremely dissatisfied, 2 = dissatisfied, 3 = neutral, 4 = satisfied, 5 = extremely satisfied. LMHA = local mental health authority. N = number of employees providing ratings. SD = standard deviation.

**Table 6:** Champion Ratings of TTTF-provided Curriculum and Training.

LMHA		“Overall, Please Rate Your Satisfaction with Curriculum Provided to You by the TTTF Team”	“Overall, Please Rate Your Satisfaction with Training You Received to Implement the Curriculum Provided to You by the TTTF Team”
	N	Mean (SD)/Median [IQR]	
All LMHAs	10	4.78 (0.44)/5 [5]	4.80 (0.42)/5 [5]
LMHA 1	4	4.75 (0.50)/5 [4.5–5]	4.75 (0.50)/5 [4.5–5]
LMHA 2	3	5.00 (0.00)/5 [5]	4.67 (0.58)/5 [4, 5]
LMHA 3	3	4.67 (0.58) /5 [4, 5]	5.00 (0.00)/5 [5]

Note. The scale for these items was 1 = extremely dissatisfied, 2 = dissatisfied, 3 = neutral, 4 = satisfied, 5 = extremely satisfied. LMHA = local mental health authority. N = number of participating champions. SD = standard deviation. IQR = interquartile range.

## Data Availability

The data presented in this study are available on request from the corresponding author. The data are not publicly available due to privacy and confidentiality concerns given the very small group LMHAs of participating champions, facilitating the ability to link champions with their data, which could affect dynamics of ongoing work relationships in unknown ways.

## Supplementary Materials

The following are available online at https://www.mdpi.com/article/10.3390/ijerph182111635/s1, Table S1: Participating Local Mental Health Authority and Champions’ Pre-Program Implementation Demographic Information.
