# Supplementary material for: Implementation and Outcomes of a Train-the-Trainer Program at Behavioral Health Treatment Centers as a Mechanism to Maintain Organizational Capacity to Address Tobacco Use Disorder
Source: Int J Environ Res Public Health. 2021 Nov 5;18(21):11635. doi: 10.3390/ijerph182111635 (PMC8583380; doi:10.3390/ijerph182111635)
Supplement: Supplementary file 1 [file ijerph-18-11635-s001.zip › ijerph-1398227-supplementary.pdf]

Supplemental Table S1. Participating Local Mental Health Authority and Champions' Pre-Program Implementation Demographic Information.

	Total Annual Patient Contacts	Unique Patients Served Annually	Number of Individual Clinics	Number of Full-time Employees	Number of Full-time Providers	Counties Served (% Rural)	Male Champions (N/%)
LMHA 1	92,498	5,420	42	247	150	23 (100%)	1 (25.0%)
LMHA 2	229,482	9,808	20	323	254	4 (50%)	1 (33.3%)
LMHA 3	239,672	11,243	31	419	286	6 (88.3%)	0 (0%)
